# Artificial Intelligence Advancements in the Cardiovascular Imaging of Coronary Atherosclerosis

**DOI:** 10.3389/fcvm.2022.839400

**Published:** 2022-03-21

**Authors:** Pedro Covas, Eison De Guzman, Ian Barrows, Andrew J. Bradley, Brian G. Choi, Joseph M. Krepp, Jannet F. Lewis, Richard Katz, Cynthia M. Tracy, Robert K. Zeman, James P. Earls, Andrew D. Choi

**Affiliations:** ^1^Division of Cardiology, The George Washington University School of Medicine, Washington, DC, United States; ^2^Department of Internal Medicine, The George Washington University School of Medicine, Washington, DC, United States; ^3^Department of Radiology, The George Washington University School of Medicine, Washington, DC, United States

**Keywords:** artificial intelligence, machine learning, coronary artery disease, cardiovascular imaging, atherosclerosis

## Abstract

Coronary artery disease is a leading cause of death worldwide. There has been a myriad of advancements in the field of cardiovascular imaging to aid in diagnosis, treatment, and prevention of coronary artery disease. The application of artificial intelligence in medicine, particularly in cardiovascular medicine has erupted in the past decade. This article serves to highlight the highest yield articles within cardiovascular imaging with an emphasis on coronary CT angiography methods for % stenosis evaluation and atherosclerosis quantification for the general cardiologist. The paper finally discusses the evolving paradigm of implementation of artificial intelligence in real world practice.

## Introduction

Artificial intelligence (AI) is a broad term that refers to computing that can perform complex human-like tasks (1). Machine learning (ML) is a subset of AI which encompasses a growing collection of algorithms that is divided into supervised learning, unsupervised learning, semi supervised learning, and reinforcement learning (2, 3). Supervised learning refers to learning from labeled examples, while unsupervised and reinforcement learning performs unlabeled learning and learning from pattern recognition (4). Further subsets of ML include Deep Learning (DL), which uses complex data sets which mirror human neural networks (5). Human cognition is finite, but the use of AI may allow for improved discrimination and evaluation of these immense datasets (6). At the same time, the “black-box” nature of AI can lead to uncertainty in clinical practice in part due to the complexity of the algorithms, unrecognized bias and application to appropriate clinical needs (7).

The application of AI in cardiology has increased exponentially annually, specifically in the diagnosis of coronary artery disease (CAD). These novel approaches may enhance future implementation of the new 2021 American College of Cardiology/American Heart Association Chest Pain Guideline that elevate the role of imaging to Class I recommendation in both acute and stable chest pain in intermediate risk patients (8). Current investigations aim to augment and enhance current risk-based approaches through the analysis of multiomic data sets, while also recently showing promise in the direct image interpretation of cardiac and coronary structures through a myriad of approaches (9). Between 2001 and 2015 the proportion of AI/ML related articles in relevant journals per month was 0.1% in relation to the total number published in the journals. This increased to 16.2% per month by 2020 (1). Literature search was completed on PubMed and EMBASE databases by searching “artificial intelligence” or “machine learning” AND various terminology related to cardiology such as “cardiac,” “cardio,” “cardiology,” “infarct,” “valve,” “cardiosurgical,” etc. (1). Over the past 5 years there were over 3,000 papers published in Pubmed related to AI/ML learning in cardiovascular medicine (4). In cardiovascular imaging specifically, there's been increases in non-invasive coronary imaging including applications of coronary artery plaque, automated calcium scoring, perivascular fat attenuation and machine learning based image enhancement as well in the application of nuclear imaging, and myocardial perfusion to large data sets to improve risk enhancement. This review article serves to summarize the recent advancements (Table 1) of Artificial Intelligence in respect to coronary artery disease (CAD) and imaging.

**Table 1:** Summary of high-yield artificial intelligence/machine learning studies in coronary artery disease imaging.

**Study**	**Population**	**Method**	**Application**	**AI method**	Performance*
Griffin et al. (10)	Diverse stable chest pain patients from 23 global sites undergoing CCTA plus quantitative coronary angiography, stress testing and fractional flow reserve (CREDENCE study)	Direct image analysis using a series of validated convolutional neural networks for AI-guided evaluation of coronary segmentation, lumen wall evaluation and plaque characterization of CCTA images	Ground truth: Core-Lab quantitative coronary angiography and invasive fractional flow reserve for identification of % coronary stenosis and adverse plaque characteristics in comparison to in	Validated convolutional neural network models; Image analysis 10 mins	Accuracy, sensitivity, specificity of 86%, 94%, 82% for ≥70% stenosis. Intra-class correlation of 0.73; For false positive AI-CCTA (≥70% by AI-CCTA, QCA <70%), 66% of vessels had FFR <0.8
Choi et al. (11)	Acute and stable chest pain patients from 3 international centers undergoing CCTA (CLARIFY study)	Direct image analysis using a series of validated convolutional neural networks for AI-guided evaluation of coronary segmentation, lumen wall evaluation and plaque characterization of CCTA images.	Ground truth: Level 3 Expert consensus for identification of % coronary stenosis and adverse plaque characteristics	Validated convolutional neural network models; Image analysis 10 mins	Accuracy, sensitivity, specificity for ≥70% stenosis was 99.7, 90.9, 99.8%. Mean difference for maximal diameters stenosis −0.8% (95% CI 13.8% to −15.3%)
Nakanishi et al. (12)	Asymptomatic adults without known CHD, part of CAC Consortium, *n* = 66,636	Coronary artery calcium and clinical variables. 77 variables incorporated, including ASCVD risk score, age, sex, race, CACS, and the number, volume and density of CAC plaques	Risk prediction for ASCVD related death and CHD related death	ML using a 10-fold cross validation framework to train and evaluate the model, as well as information gain ratio and model building using an ensemble algorithm	AUC 0.845 and 0.860 for ML predicting CVD death and CHD death respectively, compared to 0.821 and 0.835 for clinical data alone, and 0.781 and 0.816 for CAC score alone
Al'Aref et al. (3)	Stable patients with suspected CAD, from CONFIRM registry, *n* = 13,054	Coronary artery calcium and clinical variables. 25 clinical variables used, including age, gender, diabetes mellitus, hypertension, cholesterol levels	Prediction of obstructive CAD on CCTA	ML using a gradient boosting algorithm. A ten-fold cross validation framework was used to train and evaluate the model	AUC 0.881 for ML + CACS, compared to ML alone (0.773), CAD consortium clinical score (0.734), and with CACS (0.866)
Hu et al. (13)	Stable patients with suspected CAD from the REFINE SPECT registry, *n* = 1980	Stress/rest ^99m^Tc-sestamibi/ tetrofosmin MPI with SPECT, followed by invasive coronary angiography within 6 months. 18 clinical, 9 stress test, and 28 imaging variables utilized	Early coronary revascularization (ECR) prediction for stable patients after stress testing	ML using a ten-fold cross validation framework to train and evaluate the model, as well as information gain ratio and model building using an ensemble LogitBoost algorithm	AUC of ECR prediction by ML (0.812)
Oikonomou et al. (14)	Patients with stable chest pain referred for CCTA, *n* = 1575	CCTA, including perivascular adipose tissue data, and clinical variables.	5-year MACE risk prediction (cardiac death, non-fatal MI, late revascularization, non-cardiac death)	ML using random forest algorithm and repeated five-fold cross-validation	MACE prediction with and without addition of perivascular adipose tissue data (AUC 0.880 vs. 0.754)
Betancur et al. (15)	Patients who underwent clinically indicated exercise or pharmacologic stress myocardial perfusion SPECT imaging, *n* = 2,619	Rest/stress 1-day ^99m^Tc-sestamibi imaging. 28 clinical variables, 17 stress test variables, and 25 imaging variables used.	3-year MACE risk prediction, including all-cause mortality, non-fatal myocardial infarction, unstable angina, or late coronary revascularization	ML using a ten-fold cross validation framework to train and evaluate the model, as well as information gain ratio and model building using an ensemble LogitBoost algorithm	MACE prediction by ML (AUC 0.81), vs. automated stress TPD (0.73) and physician interpretation (0.64)
Motwani et al. (16)	Stable patients with suspected CAD, from CONFIRM registry, *n* = 10,030	Clinical and CCTA data. 25 clinical and 44 CCTA parameters evaluated, including segment stenosis score, segment involvement score, number of segments with non-calcified, mixed or calcified plaques, age, sex, gender, and FRS	Risk prediction of 5-year all-cause mortality of CAD	ML using a 10-fold cross validation framework to train and evaluate the model, as well as information gain ratio and model building using an ensemble algorithm	AUC 0.79 for ML predicting 5-year all cause mortality vs. FRS (0.61) and CCTA severity score (0.64 for SSS)
Arsanjani et al. (17)	Stable patients with suspected CAD, *n* = 713	Rest^201^Thallium/stress ^99m^Technetium with SPECT, followed by invasive coronary angiography within 3 months. 33 total clinical, stress test, and imaging variables utilized.	Early coronary revascularization prediction for stable patients after stress testing	ML with model building using an ensemble LogitBoost algorithm and a ten-fold cross validation framework to train and evaluate the model	Receiver operator characteristic AUC of 0.81 for ML, vs. 0.81 for reader 1, 0.72 for reader 2, and 0.77 for standalone measure of perfusion
Kang et al. (18)	Patients who underwent clinically indicated CCTA, *n* = 42	CCTA patient datasets, with visual identification of lesions with stenosis ≥25% by three expert readers, using consensus reading	Automated CCTA reading to detect both obstructive (stenosis ≥50%) and non-obstructive (stenosis 25–50%) CAD.	ML incorporating a learning-based method and an analytic method. A ten-fold cross validation framework was used to train and evaluate the model	Receiver operator characteristic AUC of 0.94 for detecting obstructive and non-obstructive lesions

*All values statistically significant, p <0.05.

*ML, machine learning; AUC, area under curve; CACS, coronary artery calcium score, ASCVD, atherosclerotic cardiovascular disease; CHD, coronary heart disease; CCTA, coronary computed tomography angiography; CAD, coronary artery disease; FRS, Framingham risk score; SSS, segment stenosis score; FRP, fat radiomic profile; MPI, myocardial perfusion imaging; ECR, early coronary revascularization; TPD, total perfusion defect*.

## Coronary Artery Disease Risk Prediction

Current prevention guidelines incorporate the use of the pooled cohort equation for adults aged 40–75 with non-traditional cardiovascular risk factors to determine a 10 year risk of cardiac events (19). The pooled cohort equation creates a simplified risk score with a finite number of variables; however the simplification of the approach may overestimate cardiovascular risk in certain populations (20, 21). Multiple studies have sought to enhance the risk prediction model. AI has been particularly impactful in the area of improved cardiovascular risk prediction (16). Nakanishi et al. demonstrated that ML using logistic regression modeling that incorporate multiple clinical and cardiac computed tomography (CT) variables was superior in predicting 10 year coronary artery disease death [area under the curve (AUC) = 0.86] than clinical data alone (AUC = 0.835), coronary artery calcium (CAC) alone (AUC = 0.816), or ML CT (AUC = 0.827) (12). This model used a total of 77 variables [46 clinical such as atherosclerotic cardiovascular disease (ASCVD) risk score, sex, age] and 31 CT variables from CAC scan to train the machine learning algorithm. Similarly, Motwani et al. (22) demonstrated that ML incorporating 25 clinical and 44 coronary computed tomography angiography (CCTA) parameters better predicted 5 year mortality than current clinical or imaging metrics. This study involved 10,030 patients, and ML exhibited a higher AUC compared with Framingham Risk Score (FRS) (ML 0.79 vs. FRS 0.61) or CCTA severity scores such as segment stenosis score (SSS) (ML 0.79 vs. SSS 0.64) (22). Al'Aref studied 13,054 patients in The Coronary CT Angiography Evaluation For Clinical Outcomes (CONFIRM) registry. ML with CAC performed the best in predicting obstructive CAD on CCTA (AUC 0.881) compared to ML alone (AUC = 0.682) and CAD consortium clinic score +CACS (0.866) (3). These models demonstrate the potential to improve predictive models. By incorporating numerous variables, both clinical and imaging, these machine learning algorithms can better predict cardiovascular mortality. Therefore, in addition to current tools such as the 10-year ASCVD risk, CAC score, and CCTA, these models can prove to add invaluable information in assessing a patient's cardiovascular risk.

## Coronary Stenosis

The recent 2021 American Heart Association/American College of Cardiology/Multisociety Guideline for the Evaluation and Diagnosis of Chest Pain redefines the presence of coronary artery disease as obstructive (≥50% stenosis) and non-obstructive (<50%) stenosis (23). In the realm of invasive and non-invasive coronary angiography (*via* CCTA), the evaluation of coronary artery stenosis requires visual analysis by a trained provider (18, 24). However, there can be inter-provider variability in interpretation of these studies in real world practice. Lu et al. (25) showed in the analysis of the Prospective Multicenter Imaging Study for Evaluation of Chest Pain (PROMISE) trial, coronary CTA scans read by both core laboratory and local site readers, 41% of the scans were in discordance regarding the presence of significant stenosis (defined as stenosis ≥50%). There remains great interest in identifying solutions that allow for improved reproducibility. AI has shown promising advancements in the detection of obstructive CAD. In 2015, Kang et al. (26) demonstrated that machine learning algorithms allowed for the detection of both obstructive (≥50% stenosis) and non-obstructive (<50% stenosis) lesions with an AUC of 0.94. In addition, Freiman et al. (27) used a deep sparse autoencoder—mixed structure regularization approach in 90 subjects and observed an AUC that ranged from 0.78 to 0.94 for discrimination of mild stenosis <30% to severe stenosis ≥70%.

**Figure 1:**
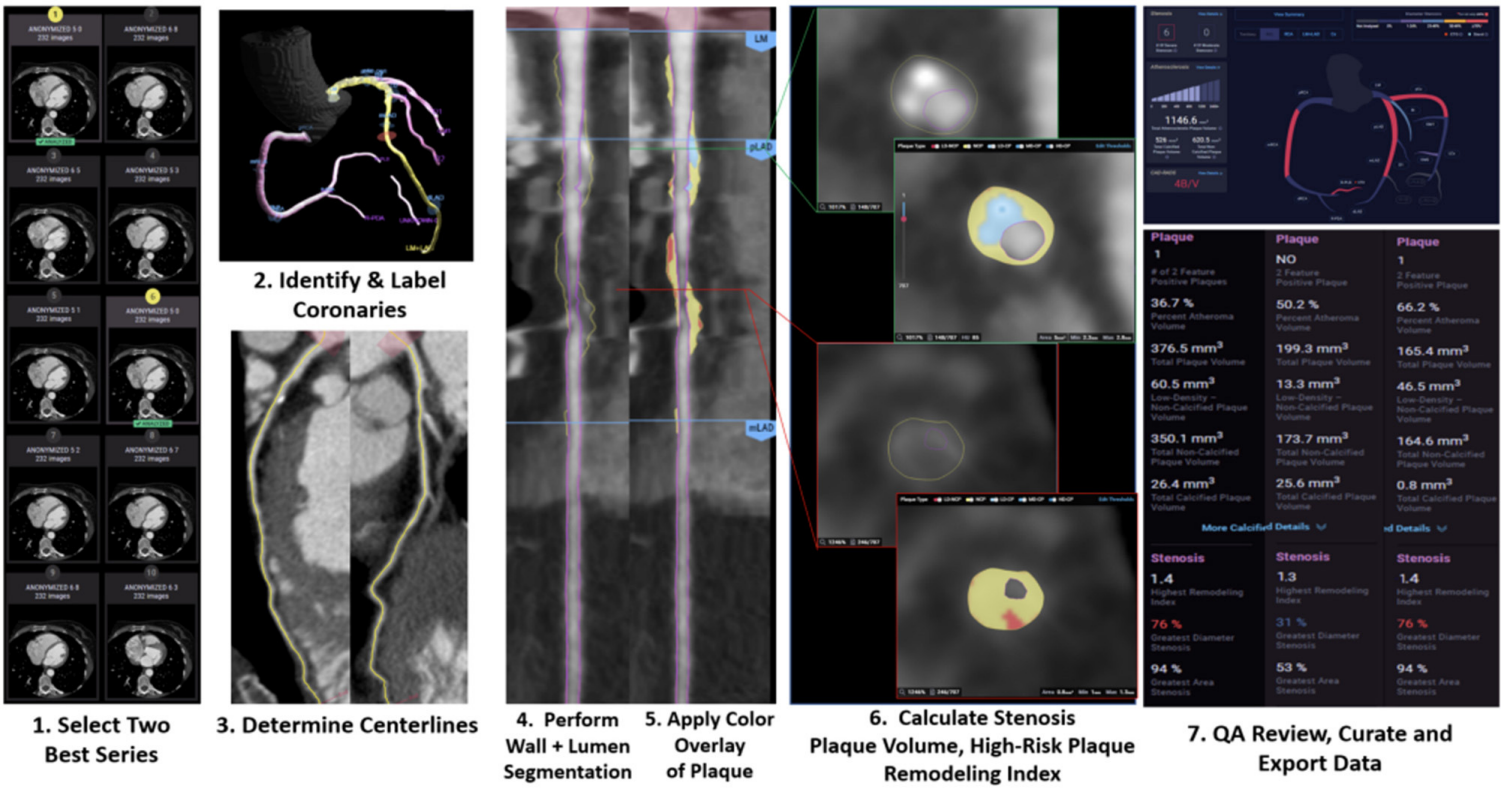
Case example of AI-guided coronary computed tomography angiography. Using a series of validated convolutional neural network models (including VGG19 network, 3D U-Net, and VGG Network variant) for image quality assessment, the machine learning algorithm (Cleerly, New York, NY) selects the best series, identifies and labels all of the major epicardial coronaries and their side branches, determines centerlines, performs coronary segmentation and labeling and then performs a rapid assessment of % stenosis, plaque volume and of adverse plaque characteristics. The data is then displayed in a graphical output to allow for clinical review.

More recently, the CT Evaluation by Artificial Intelligence for Atherosclerosis, Stenosis and Vascular Morphology (CLARIFY) multi-center study compared AI to level 3 (L3) readers in detecting coronary artery stenosis on CCTA (11). AI analysis (Figure 1) showed 99.7% accuracy in detecting >70% stenosis and 94.8% accuracy in detecting >50% stenosis. Among the vessels analyzed, the mean difference in maximal diameter stenosis between AI and L3 readers was minimal at −0.8 %. AI analysis to determine the Coronary Artery Disease Reporting and Data System (CAD-RADS) categorization compared to L3 readers was also examined. AI generated a CAD-RADS score that was in agreement with the readers in 78% of scans, and generated a score that was in agreement within 1 category in 98% of the scans. A subsequent analysis by Griffin et al. evaluated a multi-center cohort of patients undergoing core-lab quantitative invasive angiography (QCA) and found that AI CCTA had high diagnostic accuracy when compared to QCA in detecting >50% stenosis (AUC 0.88) and >70% stenosis (AUC 0.92) (10). The analysis time was approximately 10 mins, which represents an improvement over the several hours that previous methods have required. The deep convolutional neural network based approach utilized in these studies has been cleared by the Food and Drug Administration (FDA), and is clinically available and are expected to enable widespread generalizability of the studied approaches.

## Coronary Atherosclerosis Quantification

Beyond coronary stenosis or coronary artery calcium contemporary evidence has shown that the quantification of adverse atherosclerotic plaque characteristics enhances prognostication of patients at elevated risk for acute coronary syndrome (ACS) (28). Rosendael et al. (29) showed that calcium density (calculated from semi-automated software) can be associated with ACS risk. In this study, patients with and without ACS had similar calcified plaque volume. However, those who experienced ACS had less highly dense plaque, termed by the authors as “1K plaque” (HU > 1,000) than ACS-free subjects, suggesting that 1K plaque has lower risk for acute plaque rupture. The SCOT-HEART trial showed that low-attenuation plaque (HU <30) was associated with three times the risk of coronary heart disease death or nonfatal MI (30). Other features of plaques which contribute to the prognosis of CAD were studied by Yang et al. (31). The investigators used machine learning to analyze vessels in CCTA that had low fractional flow reserve (FFR) ( ≤ 0.80). In these vessels, adverse plaque features beyond lumen area which were found to be associated with low FFR vessels that included: percent atheroma volume, fibrofatty/necrotic core volume, plaque volume, proximal left anterior descending coronary artery involvement, and remodeling index. Al' Aref et al. (32) trained a machine learning model to detect culprit lesions (which had been confirmed on invasive coronary angiography) by combining quantitative and qualitative plaque features on CCTA. The machine learning model yielded an area under the curve of 0.77 for identifying the culprit lesion, significantly outperforming other models that were based solely on diameter stenosis or high-risk plaque features.

Quantification of plaque and identifying high risk plaque features is time consuming, often taking several hours for a single study, and requires a high level of expertise in a dedicated research core lab limiting such application to real world practice (33). Machine learning offers tremendous potential to allow for interpretation of imaging matrices that encompass the millions of pixels required to fully quantify atherosclerotic plaque from CCTA data in the clinical world. The aforementioned CLARIFY study by Choi et al. and the subsequent evaluation by Griffin et al. has evaluated a broad range of atherosclerosis plaque features using AI analysis (11, 34). Subsequent initial analysis has further shown that AI detected high risk features, such as lumen volume and low-attenuation, more often than experienced level 3 readers as well as accuracy when compared to fractional flow reserve. Furthermore, the analysis was performed as little as under 10 mins.

There are a number of important ongoing applications of plaque quantification. Budoff et al. (35) demonstrated that in CAD patients with elevated triglyceride levels and already taking a statin, icosapent ethyl significantly decreased the volume of low-attenuation plaque compared to placebo over an 18 month period. The application of well-validated AI guided approach to atherosclerosis quantification may enable important advances in assessing the response to preventive therapies.

## Nuclear Myocardial Perfusion Imaging

AI has also been applied to the field of nuclear medicine (36). Its utility has been demonstrated in the evaluation of coronary artery disease via single photon emission computed tomography (SPECT). Hu et al. studied 1980 patients with suspected CAD (37). Those patients underwent SPECT imaging and later invasive coronary angiography. ML utilized multiple clinical, imaging, and stress test variables to predict the need for early coronary revascularization (ECR). On a per vessel basis, ML better predicted the need for ECR (AUC = 0.79) vs. Regional Stress Tissue perfusion deficit (TPD) (AUC = 0.71), combined view TPD (AUC = 0.71), or ischemic TPD (AUC = 0.72). This was also true on a per patient basis (AUC = 0.81). Interestingly, ML also outperformed expert nuclear readers on the need for ECR in a per patient basis. Arsanjani et al. showed that machine learning can improve the accuracy of SPECT in identifying significant CAD (≥70% stenosis). AI performed with similar, if not better, accuracy (87%) in detecting these lesions compared to two expert readers (86 and 82%) (38). A similar study found that support vector machines algorithm was superior to two expert readers in detecting obstructive CAD (AUC 0.92 vs. 0.87 and 0.88) (39). Betancur et al. also showed that compared to current clinical method (total perfusion deficit), deep learning was able to predict obstructive CAD with more accuracy per patient (AUC 0.80 vs. 0.78) and also per vessel (AUC 0.76 vs. 0.73) (14). While identifying obstructive CAD is certainly important, what is of greater clinical value is predicting those patients who will go on to have adverse outcomes. In one study, machine learning demonstrated superiority to visual analysis by physicians in predicting 3-year major adverse cardiovascular events (MACE) (AUC 0.78 vs. 0.65). When incorporating clinical information (age, gender, risk factors, family history) into the algorithm, machine learning performed with even greater accuracy in predicting MACE (AUC 0.81) (40).

Furthermore, Alonso et al. showed that by analyzing SPECT data, a machine learning model outperformed logistic regression in predicting cardiac death (AUC 0.83 vs. 0.76) (13). In analyzing patients with obstructive CAD, AI has also proven its ability to predict those that may require intervention in the future. Arsanjani et al. (41) explored the utility of AI in predicting the need for revascularization. The researchers discovered that by incorporating clinical parameters such as age, smoking history, hypertension, diabetes, and family history, machine learning algorithm could predict the need for revascularization with similar or better accuracy compared to two expert readers (AUC 0.81 vs. 0.81 and 0.72). As AI interpretion of nuclear imaging continues to improve, its clinical value may increase the automated identification of ischemia beyond currently available perfusion mapping.

## Coronary Flow

In 2011, the non-invasive evaluation of fractional flow reserve by computed tomography (FFRCT) was introduced into the field of cardiac imaging by the DISCOVER-FLOW trial (42). Machine learning has been subsequently applied to the analysis of non-invasive coronary flow (43). The MACHINE registry was the first study comparing CT FFR from machine learning algorithm vs. CT FFR from computational fluid dynamics (CFD) algorithm (15). The study demonstrated that machine learning CT FFR algorithm distinguished functionally significant obstructive CAD equally well as FFR derived from a hybrid CFD approach. Tesche et al. (44) found that machine learning CT FFR had a per-lesion sensitivity of 79% and specificity of 94% in detecting lesion-specific ischemia. The area under the curve for detecting lesion-specific ischemia was 0.89 for machine learning CT FFR, equal to CFD CT FFR (AUC 0.89) and significantly higher than CCTA (AUC 0.61) and quantitative coronary angiography (AUC 0.69). The diagnostic value of machine learning CT FFR was also studied by Dugua et al. (17), who retrospectively investigated patients with symptoms of ACS who were worked up with CCTA followed by invasive coronary angiography. The investigators identified non-culprit lesions (≥ 25% stenosis, not intervened on during invasive coronary angiography). In an average of 19.5 month follow-up period, 14 patients (29%) suffered a MACE due to these non-culprit lesions. The mean FFR CT for these non-culprit lesions was 0.78, therefore showing that FFR CT ≤ 80% in patients with symptoms consistent with ACS can be a predictor of future MACE. These studies show the clinical utility of machine learning FFR CT, which also has the potential to be more efficient. Calculating FFR CT using computational flow dynamics is technologically demanding and can take up to 10 mins (15). On the other hand Itu et al. (45) demonstrated how machine learning models could generate FFR CT in as little as 2.4 s. Mannil et al. (46) conducted a proof-of-concept study showing that machine learning and texture analysis of low-dose cardiac CT was able to detect myocardial infarction that was not visible to radiologists.

## Perivascular Adipose Inflammation by CT

Vascular inflammation is a significant contributor to atherosclerosis and plaque destabilization (47). Perivascular Adipose Tissue (PVAT) can be monitored by CT fat attenuation to predict coronary artery disease due to the inflammatory effects from the vessels to the PVAT (48, 49). Higher fat attenuation index (FAI) is associated with increased cardiovascular mortality (50). One study found that there is higher FAI in culprit lesions compared to non-culprit lesions in ACS (51, 52). Oikonomou et al. (53) studied the use of ML in PVAT in three different studies/methodologies for enhanced cardiac risk prediction beyond looking at the coronary vessel anatomy and risk factors. The first study analyzed 167 patients undergoing cardiac surgery. PVAT was biopsied for transcriptional factors and CT scan to image the PVAT was performed. This demonstrated a non-invasive method of detecting and adipose tissue microvacuolar remodeling by correlation with increased levels of Collagen Type 1 Alpha 1 Chain (53). Study 2 analyzed the Fat Radiomic Profile (FRP) of 1,575 patients from the SCOT-HART trial and concluded that high FRP (designated as ≥ 0.63) was associated with a 10.8 fold increase of MACE after adjustment for risk factors (53). Lastly in study 3, 44 patients with acute myocardial infarctions (AMI) underwent CT scans on admission and 6 months later. The authors found that there were higher FRP values consistent with adverse PVAT remodeling with persistence at 6 months compared to perivascular Fat attenuation index (FAI) which was present at only with initial presentation of AMI (53).

## Epicardial Fat Quantification

In multiple studies, the location of the epicardial fat, particularly in the left atrioventricular groove has been a modest predictor of obstructive CAD (54, 55). In a study by Commanduer et al. (56), deep learning to quantify epicardial adipose tissue (EAT) was compared to quantification from two expert readers. 70 patients underwent non-contrast calcium scans and correlation of EAT volumes with deep learning quantification highly correlated with expert readers *R* = 0.973 and R = 0.979; *p* <0.001. Deep learning quantification was also associated with increased non-calcified plaque on subsequent CCTA (5.7 vs. 1.8%, *p* = 0.026). Deep learning quantification was performed with a mean of 1.57 s ± 0.49 s compared to 15 mins for expert readers.

## Coronary Artery Calcium

Increased coronary artery calcium has demonstrated to have important prognostic significance across age and diverse ethnic groups (57–59). In addition, CAC now has an important guideline-level role in risk-stratification and treatment decisions of CAD for patients at intermediate risk. For example, in non-diabetic adults aged 40–75 with LDL-C between 70 and 189 mg/dl and a 10-year ASCVD risk between 7.5 and 19.9%, current guidelines encourage the use of CAC score to guide clinicians on de-risking patients or initiating intensive lipid lowering therapy (60). Typically, calcium scores are obtained from regular dose, ECG-gated chest CT's and require some degree of manual input (the provider selecting/confirming areas of calcification and the software subsequently generating a calcium score).

There has been recent interest in using artificial intelligence/machine learning to allow for the automated quantification of coronary artery calcium as well as incorporating a calculated coronary artery calcium score to improve current risk prediction models. Isgum et al. (61) demonstrated that coronary artery calcium score can also be obtained from low-dose chest CT performed for lung cancer screening in the smoking population. Investigators identified CAC with a statistical pattern recognition system, and then utilized support vector machines to correctly classify cardiovascular risk category in 82% of the subjects based on Agatston score. The accuracy of fully automated calcium scores from low-dose CT has also been evaluated in other studies. Takx et al. (62), examined automatic calcium scores derived from low-dose CT. There was good reliability between fully automated calcium scores and reference scores set by human readers (kappa 0.85). Most of the discordance was due to the automated method failing to detect calcifications in the right coronary artery. Isgum et al. (63), also yielded similar results. The study showed agreement of CVD risk category (based on Agatston score) not only between fully automatic and manual calcium scores derived from low dose CT (kappa 0.89), but also between fully automatic calcium score from low dose CT and calcium score from dedicated calcium scoring CT (kappa 0.74). Winkel et al. (64) used deep-learning software to calculate vessel-specific CAC sub-scores (right coronary artery, left main, left main, left anterior descending, and circumflex). The risk class assignment determined by AI showed agreement with that of human readers (kappa = 0.91). Given the association of smoking history with cardiovascular disease, and the abundance of lung cancer screening CT scans, the ability to automatically estimate calcium scores from such scans could provide the added benefit of identifying patients at increased cardiovascular risk.

## Ethics, Limitations and Standards of Artificial Intelligence in CAD Imaging

The ease in which ML can acquire data can present ethical dilemmas. Big data can be analyzed in minutes (11) raising issues such as proper consent and safe storage of protected health information (PHI). The “black box” nature of AI may lead to uncertainty for physicians seeking to apply these approaches to practice. Clinicians must be aware of the specific validation of AI (65) and the limitations to avoid unintended extrapolation and biased results. As automated analysis improves to better address gaps in expert level care, the field of cardiovascular imaging training may be inadvertently depleted of the “Human Neural Network” when there is overreliance on AI to create the foundations of analyzing images (66). The use of AI in cardiovascular medicine needs to be tailored to specific patient-centered goals to avoid unintended or false discoveries (9).

In the imaging of coronary artery disease, it is important to establish appropriate ground truth standards such as quantitative coronary angiography, fractional flow reserve and invasive ultrasound. In addition, new artificial intelligence and machine learning based approaches should be validated in randomized controlled trials (RCT). An RCT in which one arm receives usual care and the other arm receives AI assisted care can be extremely influential and may be a novel trial approach to further create an evidence base for the use AI in clinical practice. It is important that these tools are vetted by the Food and Drug Administration and similar regulatory bodies, through peer-reviewed studies as well as through the professional societies. The field will also need to further develop models for integration into clinical practice, its use as a clinical decision support tool as well as addressing scenarios in which the AI/ML tool disagrees with clinical readers. It is also expected there may be clinical scenarios in which the AI/ML has not been fully trained. For example, in an acute coronary syndrome, the AI/ML may be able to identify severity of stenosis and adverse plaque characteristics, but not recognize a coronary artery dissection. With its various application to cardiovascular medicine, there will be a continued and ongoing need to apply ethnical and scientific standards in AI/ML in coronary imaging.

## Conclusions

**Figure 2:**
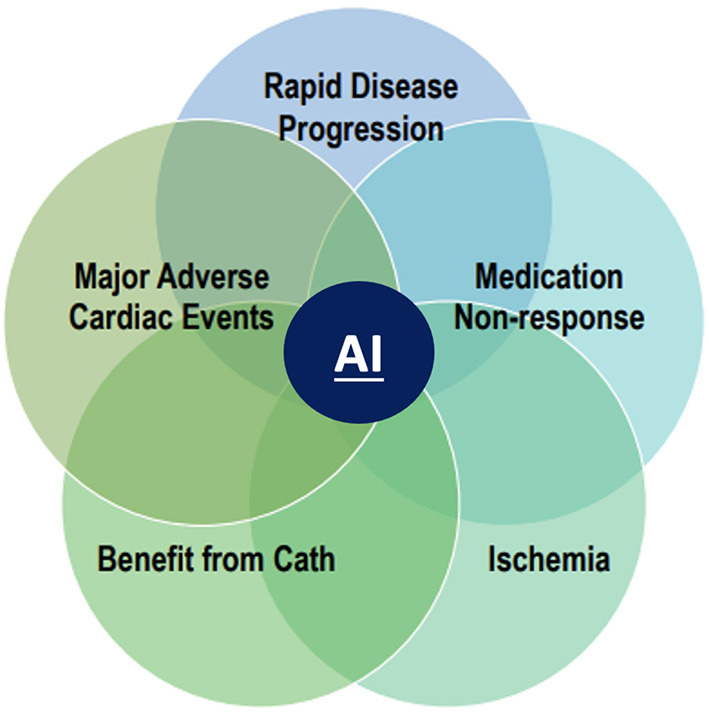
New paradigm of AI guided coronary artery disease imaging. An artificial intelligence (AI) guided approach to coronary artery disease in CAD imaging opens several new frontiers in the evaluation and treatment of atherosclerosis. These include the evaluation of rapid disease progressors, accessing response or non-response to statin and other lipid lowering therapies, improved prediction of ischemia, enhanced selection for guideline based invasive angiography and prognostication of major adverse cardiovascular events.

The promise of artificial intelligence lies in leveraging modern algorithms to improve decision making and risk prediction beyond current models that are patient-centered (Figure 2) (11). In the opinion of this author group, the recently validated stenosis and atherosclerosis quantification methods discussed in this paper, with their FDA approval and clinical availability represent a practice ready approach. Application of an AI/ML guided CCTA approach opens several new frontiers in the assessment and treatment of atherosclerosis. Specific examples include the opportunity to evaluate rapid disease progressors and those that do not respond to lipid lowering therapies. AI/ML may also allow for non-invasive evaluation of those that demonstrate plaque regression after intensified, personalized medical therapies. AI/ML guided atherosclerosis evaluation may better predict ischemia as well as those patients that will require invasive angiography

A future paradigm includes utilization of AI so that the cardiologist may use the AI/ML guided information to make improved clinical decisions and enhance patient-centered outcomes. With continued research in the field and promising outcomes it is expected that the next decade will see AI applied broadly in clinical practice to allow improved outcomes while care remains led by the cardiovascular clinician.

## Author Contributions

PC and AC contributed to the conception and design of the manuscript. PC, ED, IB, and AC wrote the first draft of the manuscript. All authors provided important intellectual review, contributed to manuscript revision, read and approved the final version.

## Conflict of Interest

JE is an employee and retains equity in Cleerly Inc. AC reports grant funding from GW Heart and Vascular Institute, and equity in Cleerly. The remaining authors declare that the research was conducted in the absence of any commercial or financial relationships that could be construed as a potential conflict of interest.

## Publisher's Note

All claims expressed in this article are solely those of the authors and do not necessarily represent those of their affiliated organizations, or those of the publisher, the editors and the reviewers. Any product that may be evaluated in this article, or claim that may be made by its manufacturer, is not guaranteed or endorsed by the publisher.
